# Crowned Dens Syndrome as a cause of acute neck pain: a Case Report and Review of the Literature

**DOI:** 10.31138/mjr.28.2.101

**Published:** 2017-06-27

**Authors:** Ali Abdul-Rahman Younis

**Affiliations:** Rheumatology Department, Faruk Medical City, Sulaymaniah, Iraq

**Keywords:** Neck pain, Calcium pyrophosphate, odontoid process, crowned dens syndrome, pseudogout

## Abstract

Crowned dens syndrome (CDS) is a rare clinical entity characterized by acute neck pain due to calcification around the odontoid process of the axis in “crown-like” configuration. Crystalline deposition in cervical vertebrae is less well known disease entity and only a limited number of cases have been reported to date. I here present a case of 79-year- old woman who developed acute severe neck pain and stiffness. Cervical computed tomography (CT) scan detected periodontoid calcification and a diagnosis of Crowned dens syndrome was made. Crowned dens syndrome should be considered in the differential diagnosis of acute neck pain, particularly in old age patients.

## INTRODUCTION

Crowned dens syndrome (CDS), also known as acute pseudogout of the cervical spine, is a radioclinical entity defined by the radiographic calcifications in a crown-like configuration around the odontoid process, accompanied clinically with acute neck pain in cervico-occipital area, often with neck stiffness, fevers and raised inflammatory markers. ^1^ This syndrome was first described by Bouvet et al in 1985. ^2^ It was postulated that the crowned dens deposits could be attributed to calcium pyrophosphate dihydrate (CPPD) crystals as well as hydroxyapatite crystals. ^3^ The spontaneous duration of these clinical manifestations is very variable, from a few days to several weeks. These crystalline deposits, most often CPPD crystals, can remain asymptomatic or be responsible for chronic neck pains or spinal cord compression. ^4^ The aim of this report is to highlight CDS as an important differential diagnosis in patients presenting with acute neck pain.

## CASE REPORT

**Figure 1:**
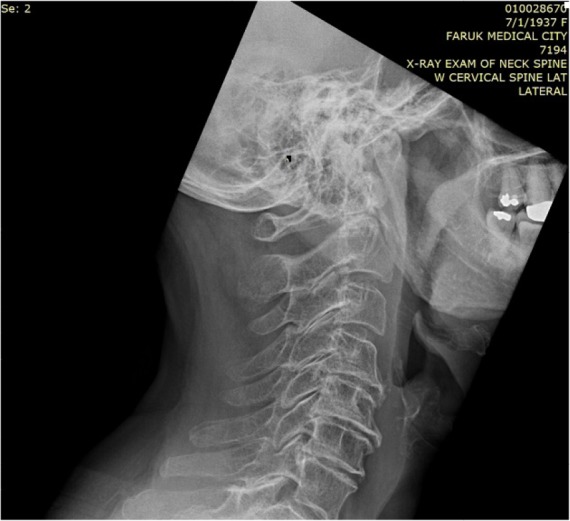
Cervical radiograph: spondylosis.

**Figure 2:**
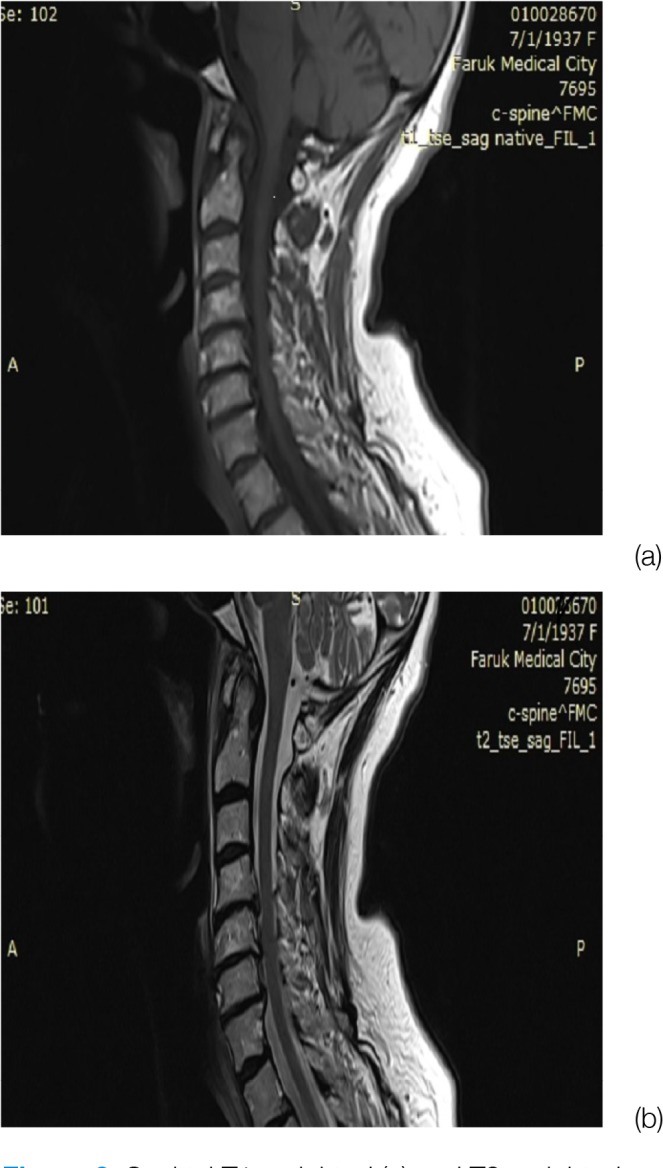
Sagittal T1-weighted (a) and T2-weighted (b) Cervical MRI: soft tissue thickening posterior to the dens.

**Figure 3.**
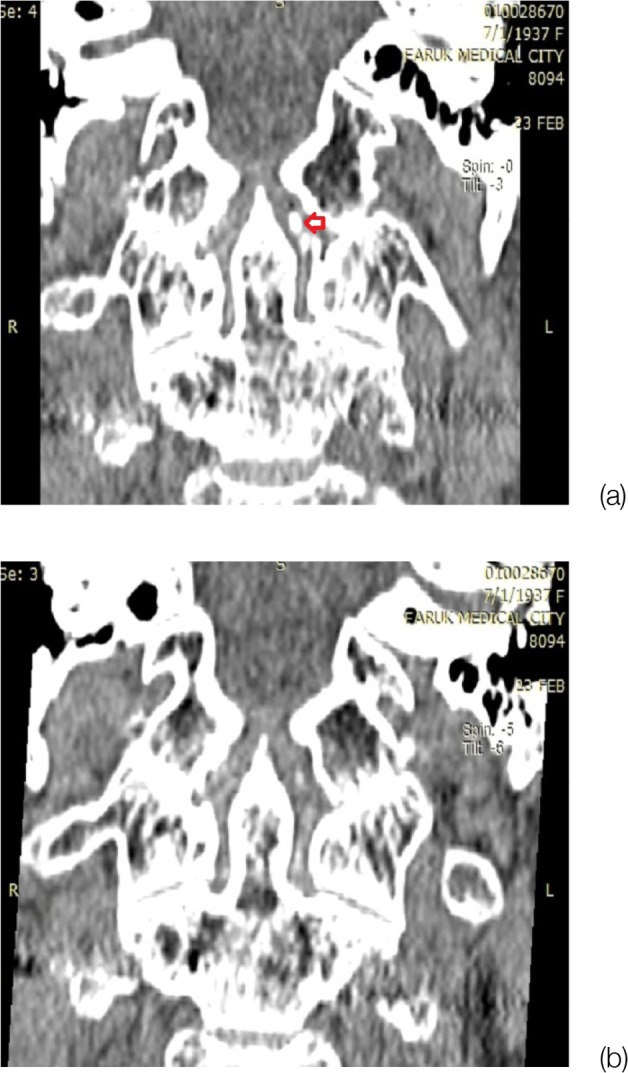
**
(a) and 3 (b):** Cervical CT: calcification lateral to the dens (coronal).

**Figure 4:**
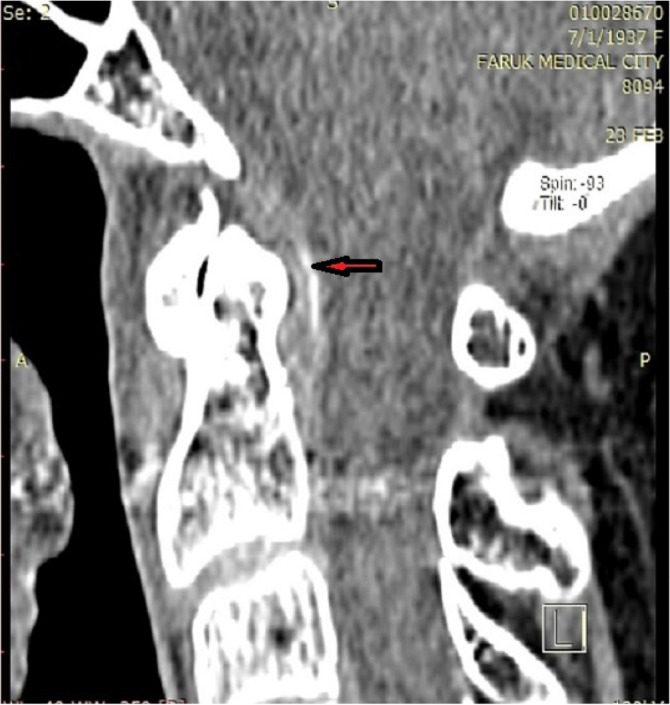
Cervical CT: linear calcification posterior to the dens (sagittal).

**Figure 5:**
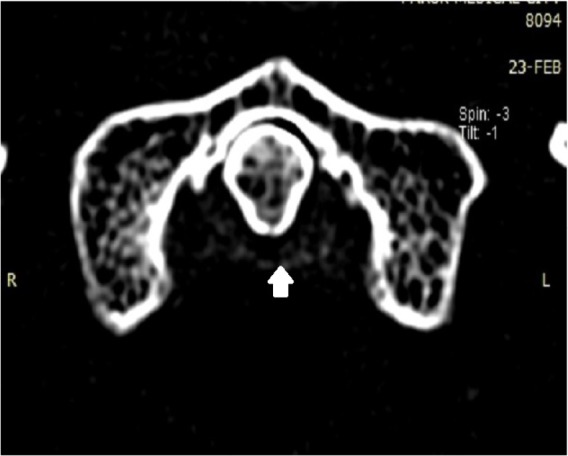
Cervical CT: calcification of the transverse ligament (axial).

A 79-year-old woman, known case of hypertension, present to the emergency department of our hospital with acute onset of severe posterior neck pain, occipital headache and fever. The patient also had neck stiffness, vertigo, and vomiting. She was admitted to the medical ward and neurological consultation made suspected meningitis. A computed tomography scan of the head was performed, in which serious pathology was excluded. Neurological evaluation was unremarkable, and rheumatologist opinion was sought. The patient admitted long history of neck pain, which was localized, mild, intermittent, and related mainly to activity. However, two weeks before admission, the patient developed severe posterior neck pain which was acute in onset, continuous, radiating into the occipital region, worst at night and associated with vertigo. She experienced gradual worsening of pain and marked restriction of neck motion. The patient reported no weight loss, myalgia, arthralgia, morning stiffness, jaw claudication or visual symptoms. The patient had no prior history of traumatic injury and no apparent history of peripheral joint pain or swelling, apart from mild knee pain with walking and climbing stairs. The patient’s family history was not contributory. Her neck pain improved partially with nonsteroidal anti-inflammatory drug which was used for few days only. On examination the patient was conscious, alert, fully oriented. She looks pale, ill. Vital signs were stable apart from temperature which was 37.8 °C. The superficial lymph nodes were not palpable. Furthermore, edema formation or skin eruption was not noted in her extremities and temporal artery was normal on palpation. Cardiac, respiratory, and abdominal examinations were unremarkable. In terms of neurological evaluation, overall cranial nerve, motor, and sensory nervous systems were intact with no detectable focal neurological deficit and negative Kernig’s sign. On local examination, there was severe cervical rigidity, diffuse tenderness over the spine and paraspinal muscles, most marked in the nape of the neck with severe muscle spasm. Passive cervical spine movements were significantly reduced. Range of neck motion was limited to 10 degrees on rotation and lateral flexion whereas extension and flexion were 20 degrees each. At the end of the range of neck motion, pain was markedly provoked. The peripheral joints were not tender or deformed, apart from knees which showed medial joint line tenderness, crepitus, and mild painful limitation of the range of motion. Regarding the laboratory data, the value of the C-responsive protein was 5.28 mg/dL (normal range, 0.0–0.5 mg/dL); leukocyte was 13600/mm3, 86% neutrophils, erythrocyte sedimentation rate was 21 mm/hour, all of the other findings including liver, kidney, and thyroid function test, serum electrolyte, blood sugar, uric acid and urinalysis were unremarkable. Blood cultures were negative. Cervical spine radiography obtained 2 weeks prior to the admission showed only cervical spondylosis with loss of cervical lordosis ( **Figure 1** ). Recent radiograph of the knees revealed advance osteoarthritis, with no evidence of chondrocalcinosis. Based on the clinical features, pyogenic spondylodiscitis was highly suspected and cervical magnetic resonance imaging was ordered which revealed soft tissue thickening posterior to the dens, with low-intermediate signal intensity on T1 and low signal intensity onT2-weighted, with advance multilevel degenerative changes and spinal cord compression ( **Figure 2** ). A plain computed tomography (CT) scan and three-dimensional (3D)-reconstruction imaging of the cervical spine detected calcification in the soft tissue surrounding the dens and on its top on coronal reconstruction ( **Figure 3** ), linear calcification posterior to the dense of axis on sagittal reconstruction ( **Figure 4** ) and faint dotted calcifications of the trans-verse ligament of the atlas on axial imaging ( **Figure 5** ). CT imaging also revealed narrowing of the atlantoaxial joint with osteophytosis. There was no evidence of osseous destruction, lytic lesions, or fractures. A diagnosis of crown dens syndrome was made. Initially, the patient was treated symptomatically with analgesic drugs and intravenous fluids. The patient was started on colchicin 0.5 mg and prednisolone 15 mg daily, in addition to omeprazole and cinnarizine. The patient was discharged from the hospital on the next day, and her symptoms drastically improved within 7 days after initiating treatment. Three weeks later, the patient was nearly asymptomatic with marked improvement in cervical range of motion and normalization of the inflammatory markers.

## DISCUSSION

CDS was first described in 1985 by Bouvet et al. who recognized it as a clinical condition characterized by acute neck pain, accompanied by calcification around the dens of C2, due to the deposition of crystals of hydroxyapatite or calcium pyrophosphate. ^2^ Although such calcification often gives rise to no symptoms, it may be associated with acute neck pain usually reflecting a non-specific inflammatory reaction around the crystals. This is called crowned dens syndrome. Salaffi et al have found that only nine of the 49 cases of crystal deposit (18.4%), presented with neck symptoms. ^5^ There are only a limited number of cases have been reported to date. ^6^ According to Goto et al, by 2007 only 35 cases had been reported in the English language literature. ^7^ The syndrome is more common in the elderly, predominantly affecting women after the age of 60. ^8^ The onset of CDS is typically acute, but may sometimes be chronic. ^7^ Patients typically present with acute severe neck pain, with concomitant neck stiffness, often with significant restriction of cervical rotation, and possibly fever, while the inflammatory markers are often elevated. ^9^ It may be detected in up to 5% of patients over the age of 70 who present to hospital with neck pain. ^7^ The syndrome may cause chronic neck pain or even spinal cord compression. ^3,4^ Also, it has been reported that calcium pyrophosphate dihydrate crystal deposition in and around atlantoaxial joint may cause fractures of the odontoid process. ^10^

The differential diagnosis of this syndrome includes mainly meningitis, discitis, tumor, abscess, polymyalgia rheumatica and giant cell arteritis. ^11^ Interestingly, a large number of patients with CDS have developed pseudogout in other joints; ^7,12^ a fact that suggests that the syndrome is most commonly caused by calcium pyrophosphate deposition disease. ^11^ However, case series presented by other authors in which there is no correlation of CDS and crystal deposition in peripheral joints. ^13^ In addition, CDS may be the initial manifestation of crystalline deposition disease. ^14^ There have been reported cases where periodontal calcifications are noted in patients with neck pain that suffered from systemic diseases, such as seronegative spondyloarthropathy, systemic sclerosis and rheumatoid arthritis. ^15^ Furthermore, high calcium level in the blood, such as in hyperparathyroidism, is thought to predispose to the syndrome. ^16^

The exact pathophysiology of CDS remains unclear. There are several theories explaining the mechanism of CPPD deposition at atlantoaxial joint. Constantin et al postulated that CPPD deposition at the atlantoaxial joint can be explained by the presence of chondroid cells within the transverse ligament of the atlas, thus making it structurally similar to the knee meniscus; a fibrocartilaginous structure. ^17^ Another theory is that fibroblasts in cervical spine ligaments transform into chondrocytes and thus making these ligaments susceptible to calcification. ^18^ Calcium deposits around the odontoid process leads to soft tissue expansion and mechanical disruption to the anatomical structures, resulting in inflammatory reaction. ^19^

The gold standard for the diagnosis of the CDS is CT scan, ^8^ because plain radiographs usually fail to detect the periodontoid calcifications. ^7^ CT scanning focusing on C1/C2 makes it possible to identify the anatomical structures affected by CDS. If the typical tiny half-ring form of calcification behind the dens corresponds to the transverse ligament of the atlas, calcifications of other anatomical structures surrounding the top and the sides of the odontoid process are described in association with the clinical picture of CDS. ^2,4,7^ In the radiological classification of CDS proposed by Goto et al., CPPD deposition may be found posterior only (50%), postero-lateral(27.5%), circular (12.5%), lateral (5%) or anterior (5%) to the odontoid process. ^7^ According to some authors, ^17, 20, 21^ the definition of CDS should be extended to all the calcifications involving the synovial membrane, the articular capsule, the occipito-transverse ligament and/or the transverso-axial ligament that surrounds the dens. CT scanning performs better than MRI in assessing calcifications of the dens area. ^4^ Cervical MRI is generally of no benefit in making the diagnosis; however, it may be helpful in excluding discitis, myelopathy, or malignancy. The CT findings persist for about 3 months after the relief of symptoms of the syndrome. ^8^

The prognosis of CDS is generally good, and symptoms usually subside within a few weeks. ^7^ Taniguchi et al have found that most patients recover without sequelae with symptomatic therapy. ^22^

The majority of CDS patients fully recover within a week of high dose NSAIDs, corticosteroid, colchicine or combination therapy. ^1,7^ Non-steroidal anti-inflammatory drugs (NSAIDs) have been commonly used as first-line therapy for CDS. In cases without improvement using NSAIDs alone, treatment with moderate dosage of corticosteroids is recommended. ^6^

Crown dens syndrome should be considered in the differential diagnosis of acute neck pain, particularly in old age patients. Clinicians should be aware of the clinical features of CDS to prevent misdiagnosis, invasive and unnecessary investigations (lumbar puncture, biopsy), inappropriate treatment (antibiotics, antiviral drugs) and prolonged hospitalization.
